# The Effect of Electroacupuncture versus Manual Acupuncture through the Expression of TrkB/NF-*κ*B in the Subgranular Zone of the Dentate Gyrus of Telomerase-Deficient Mice

**DOI:** 10.1155/2018/1013978

**Published:** 2018-04-22

**Authors:** Dong Lin, Jie Zhang, Wanyu Zhuang, Xiaodan Yan, Xiaoting Yang, Shen Lin, Lili Lin

**Affiliations:** ^1^College of Acupuncture, Fujian University of Traditional Chinese Medicine, Minhou Shangjie, Fuzhou, Fujian 350122, China; ^2^Department of Rehabilitation, People's Hospital Affiliated to Fujian University of Traditional Chinese Medicine, Fuzhou, Fujian 350002, China; ^3^College of Integrated Traditional Chinese and Western Medicine, Fujian University of Traditional Chinese Medicine, Minhou Shangjie, Fuzhou, Fujian 350122, China

## Abstract

Our previous study showed that the acupuncture stimulation on the acupoint (ST-36) could activate the brain-derived neurotropic factor (BDNF) signaling pathways in telomerase-deficient mice. Recently, we set out to investigate whether the manual acupuncture (MA) or electroacupuncture (EA) displays a therapeutic advantage on age-related deterioration of learning and memory. Both telomerase-deficient mice (Terc^−/−^ group, *n* = 24) and wild-type mice (WT group, *n* = 24) were randomly assigned to 3 subgroups (CON, controls with no treatment; MA, mice receiving manual acupuncture; EA, mice receiving electric acupuncture). The mice were subjected to behavior test, and EA/MA were applied at bilateral acupoints (ST36) 30 min daily for 7 successive days. The brain tissues were collected after the last Morris water maze (MWM) test and were subjected to the immunohistochemistry and western blot analysis. The MWM test showed that EA can significantly increase the time in target quadrant (*P* ≤ 0.01) and frequency of locating platform for Terc^−/−^ mice (*P* ≤ 0.05), while nothing changed in WT mice. Furthermore, western blotting and immunohistochemistry suggested that EA could also specifically increase the expression of TrkB and NF-*κ*B in Terc^−/−^ mice but not in wild-type mice (*P* ≤ 0.05). Meanwhile, the expression level and ratio of ERK/p-ERK did not exhibit significant changes in each subgroup. These results indicated that, compared with MA, the application of EA could specifically ameliorate the spatial learning and memory capability for telomerase-deficient mice through the activation of TrkB and NF-*κ*B.

## 1. Introduction

Aging related neurodegeneration diseases are currently the mostly studied area in neuroscience. It is well known that aging is a multifactorial complex process that leads to the deterioration of biological functions, and the telomeres and telomerase may play a key role in this biological aging [1]. It is previously known that telomere protects chromosomes and plays important role in the cell life for its prolonged persistence. The telomerase is a DNA polymerase that plays an important role in telomere synthesis [2, 3]. In the nervous system, the neurons during embryonic and early postnatal life have high levels of telomerase activity, while in adult brain the level rapidly decreases, and at the same time the apoptosis of neurons occurs naturally during development. Therefore, some researchers believed that the reducing telomeres appear to be essential for the aging process in different organism [4, 5]. Previous research suggested that adult neurogenesis declines with age, and the age-related neurodegeneration could be due to dysfunctional telomeres, especially the telomerase with deficiency [3, 6, 7].

It is well known that acupuncture treatment taking as an traditional Chinese medicine has been widely used in some neurological disorders. Furthermore, some studies have demonstrated that acupuncture or electroacupuncture exerted a vital function in the treatment of Alzheimer disease (AD), even proven a great efficiency in improving intelligence [8]. Some researches indicated that acupuncture treatment targeted the acupoints in surface and finally resulted in particularly neuroprotective effects in nervous system [9, 10]. Among the nonpharmacological techniques, manual acupuncture (MA) and electroacupuncture (EA) were the basic two categories to acupuncture [11]. Compared with the different patterns of stimulation, EA is more repeatable and adjustable, while MA is more flexible and suitable in many diseases. Furthermore, there were more and more research that revealed that both EA and MA could improve cognitive deficits in AD animal models. Meanwhile, our previous studies have indicated that manual acupuncture could activate the brain-derived neurotropic factor (BDNF) and its downstream signaling pathways for neuroprotection [12, 13]. However, accumulating evidence has demonstrated that the therapeutic effect of EA was focused on attenuating cognitive deficits and increasing pyramidal neuron number in hippocampal [14, 15]. In the present study, we investigated the difference of effect induced by EA and MA on telomerase-deficient mice. In addition, we further explored the expression of TrkB (tropomyosin receptor kinase B)/NF-*κ*B (nuclear factor kappa-light-chain-enhancer of activated B cells)/ERK (extracellular regulated protein kinases)/p-ERK (phosphorylated-extracellular regulated protein kinases) protein in the subgranular zone (SGZ) of the dentate gyrus (DG) of telomerase-deficient mice.

## 2. Materials and Methods

### 2.1. Animals

The mice deficient for TERC genes were provided by the Jackson Laboratory in United States (Stock #004132). The experiments were approved by the Institutional Animal Care and Use Committee of Fujian University of Traditional Chinese Medicine, China, and performed according to the NIH Guideline for the Care and Use of Laboratory Animals. The mice were housed in an environmentally controlled vivarium under a 12 h light–dark cycle and temperature 23 ± 2°C, humidity 50–60%. Food and water were available for freedom usage. All the animals were generated by inbreed crossing of heterozygous knockout mice that were backcrossed to naïve C57BL/6J mice for more than 3 generations. The mice were genotyped using Polymerase Chain Reaction to confirm the genetic modifications. Two strains of 7-month-old adult mice were used for the current study (i.e., wild-type mice [WT, *n* = 24] and telomerase-deficient mice [Terc^−/−^, *n* = 24], with 8 mice each in 3 subgroups).

### 2.2. Experimental Protocol

**Figure 1:**
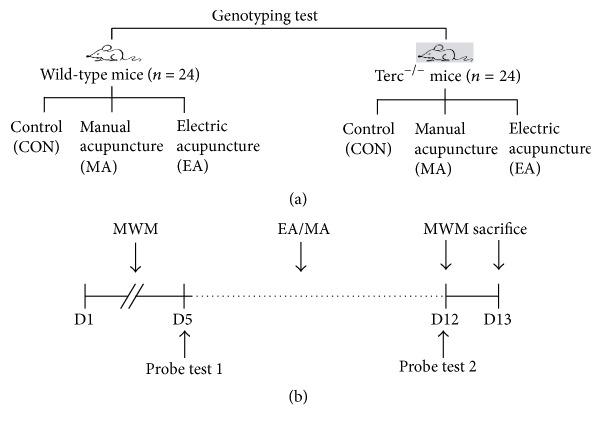
Experiment design on grouping, schematic representation of the methodology used. (a) All of the WT group mice and Terc^−/−^ group mice were randomly distributed to 3 subgroups (*n* = 8 per subgroup): (1) controls with no treatment, (2) mice receiving manual acupuncture (MA), (3) mice receiving electric acupuncture (EA). (b) The mice received MWM test. In the 5th day, the MWM test was taken, and then MA/EA treatment was performed for 7 days. After the last MA/EA treatment administration, the last probe test was carried out. And all of the mice were sacrificed after the last behavioral observations (24 h later).

The mice in both the WT group (*n* = 24) and the Terc^−/−^ group (*n* = 24) were randomly assigned to 3 subgroups, 8 mice to each subgroup per group (Figure 1): (1) the control subgroup (CON) without any treatment; (2) the manual acupuncture subgroup (MA) that received manual acupuncture at the acupoint ST-36; (3) the electroacupuncture (EA) subgroup that received an electrical acupuncture stimulation on acupoint ST-36. All animals were observed while the acupuncture was performed, and if anyone looked uncomfortable, it was stroked gently on the back until it became calm again [16].

### 2.3. Morris Water Maze (MWM) Behavioral Test

For the purpose of evaluating the ability of learning and memory, Morris water maze procedure was performed as described [17, 18]. The water maze consisted of a circular tank (120 cm in diameter, 50 cm in height) filled with water to a depth of 28.5 cm, maintained at 22 ± 2°C. The area of the pool was conceptually divided into four quadrants (NE, NW, SW, and SE) of equal size. In the center of the 3rd quadrant, we placed a hidden escape platform as the target quadrant with 12.5 cm in diameter. The mice were given 60 s to locate the hidden platform. Once the mice found the submerged platform, it could remain on it for 10 s, and the latency to escape was recorded. Any mouse that failed to locate the platform within 60 s was placed on the platform by hand. Each mouse was subjected to 4 training trials per day for 4 consecutive days. Twenty-four hours after the final trial, the assessing spatial memory was taken in probe test. In this test, the mice need to swim freely for 60 s without the platform in the tank. Time spent in the target quadrant and the frequencies of locating platform were taken to indicate the degree of memory consolidation after learning. All data were collected by a video camera (TOTA-450III, Japan) and analyzed by an automated analyzing system (Dig-Behav, Jiliang Co. Ltd., Shanghai, China). Considering the following 7-day acupuncture treatment, we designed two probe tests after 4-day training time [19]. The probe test 1 was arranged before acupuncture intervention, and the probe test 2 was carried out after the last day of treatment.

### 2.4. Acupuncture Intervention

The control subgroup did not receive any treatment but were immobilized by hand with gentle plastic restraints just as the treatment groups. In the treatment group, acupuncture stimulation was performed by a small acupuncture needle (13 mm in length, 0.3 mm in diameter, from Suzhou Hua Tuo Medical Instrument Co., Suzhou, China). Because of the effectiveness in improving the brain function, the point of bilateral ST-36 was chosen to be used. The locations of ST-36 and acupuncture manipulation were chosen following our previously described protocol [13, 14]. In the MA subgroup, the manual acupuncture on the point of ST-36 was applied for 30 mins. The needles were inserted into acupoint for a depth of 1.5–2 mm, and twirling manipulation was applied every 5 min and lasted 20 s each time. Each needle was rotated bidirectionally within 90° at a speed of 180°/s. For EA subgroup, a pair of needles were tightly tied together and inserted to bilateral ST-36 just as reported previously [20]. The needles were was also inserted into ST-36 acupoint for the same depth just as the MA group and connected to a Han's acupoint nerve stimulator (HANS, Han's Acupoint Nerve Stimulator, Model LH 202H, Beijing Huawei Ltd., Beijing, China). The parameters were as follows: sparse-dense wave with a frequency of 2/50 Hz, current of 2 mA, 30 min/stimulation, and one stimulation per day, for 7 consecutive days.

### 2.5. Tissue Preparation and Immunohistochemistry

After behavioral test and acupuncture intervention all the animals were sacrificed under 10% chloral hydrate (0.35 ml/100 g, intraperitoneal [i.p.]), and the brain tissues were collected after intracardial perfusion with saline. The brain samples were halved for each of the subjects; the left-half was separated out for protein preparation, and the right was fixed with 4% (w/v) paraformaldehyde for next immunohistochemistry analysis. The tissue blocks containing hippocampus were dehydrated and embedded in paraffin. Fixed brains were cut in 5 *μ*m sagittal sections. The sections was mounted on 0.1% polylysine reagent (Sigma) coated slides. Subsequently, the sections were dewaxed and hydrated and incubated in 0.01 mol/L of citrate buffer for antigen thermal remediation for 5 min by being treated with microwave (700 W), and then for 10 min with 3% H_2_O_2_ at room temperature, and washed in phosphate-buffered saline (PBS) for 3 × 5 min. Next, the sections were blocked in 2% BSA for 10 min and incubated with primary antibody diluent (rabbit anti-TrkB 1 : 500, Cell Signalling Technology; rabbit anti-NF-*κ*B 1 : 200, Cell Signalling Technology) for 12 h at 4°C. Then, the sections were rinsed by PBS and next incubated with secondary antibody diluent (biotinylated goat anti-rabbit IgG, diluted 1 : 1000, Vector Laboratories) for 30 min at room temperature. After wash by PBS for 3 × 5 min, the diaminobenzidine (DAB) kit (Vector Laboratories, Burlingame, USA) was used for color development for 5 min. After being redyed with hematoxylin, the brain slices were dehydrated and observed under a light microscope, BX53 (BX-51 Olympus, Tokyo, Japan), and analyzed using Image J software.

### 2.6. Western Blot Analysis

The frozen hippocampus tissues were obtained after behavior test and were homogenized on ice in 1.5 ml RIPA protein lysis buffer supplemented with 500 g PMSF. After centrifugation for 15 minutes at 12,000 ×g at 4°C, the protein in cleared supernatant was quantified and adjusted to 5 mg/ml. Equivalent amounts of protein (30 *μ*g/lane) were separated by SDS-PAGE and transferred to PVDF membranes. Membranes were blocked with 5% (w/v) bovine serum albumin in Tris-buffered saline with Tween 20 for 1 hour and then incubated with primary antibody (rabbit anti-mouse TrkB [1 : 1000], ERK [1 : 2000], P-ERK [1 : 1000], NF-*κ*B [1 : 500], Santa Cruz Biotechnology,) overnight at 4°C. The immunoblots were then incubated with goat anti-rabbit horseradish peroxidase-conjugated IgG for 2 hours at room temperature (1 : 1,000), we applied the chemiluminescent to develop the films, and the protein bands were quantified by Quantity One. The protein expression level was controlled by the protein of *β*-actin. All the data were expressed as the ratio relative after normalization to the *β*-actin levels.

### 2.7. Statistical Analysis

All data is presented as mean ± SEM for each group. For the Morris water maze test the escape latency time of the hidden platform trial was analyzed by two-way ANOVA of repeated measures, and the probe trial including escape latencies and original angle was conducted in the form of a multifactorial analysis of variance (ANOVA). The immunohistochemistry and western blot assay data were also analyzed by one-way ANOVA analysis of variance followed by LSD (equal variances assumed) or Dunnett's T3 (equal variances not assumed) post hoc test. All the analysis was performed with Prism 6.0 (GraphPad Software Inc., San Diego, USA), and the *P* values less than 0.05 were considered statistically significant.

## 3. Results

### 3.1. Effect of Electroacupuncture on Spatial Learning and Memory

**Figure 2:**
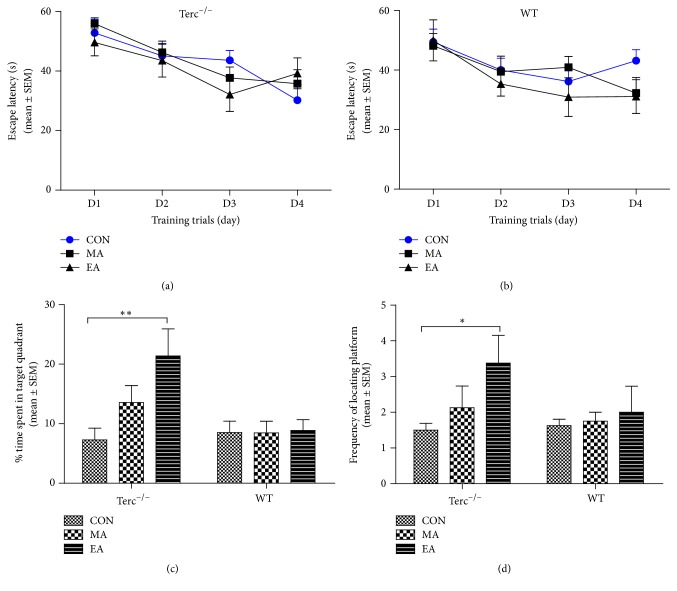
Acupuncture intervention prevented spatial and memory impairment in telomerase-deficient mice (Terc^−/−^) in Morris water maze task. (a) Performance in training trial of Terc^−/−^ mice (*n* = 8 for each subgroup) and (b) wild-type mice (*n* = 8 for each subgroup), during 4-day hidden platform trial. The data shows that escape latency to reach the hidden platform before acupuncture intervention in three subgroups. (c) In the probe test, the difference value of time spent in the target quadrant between before and after treatment was calculated. It was interesting that the EA stimulation can significantly increase the time in target quadrant for Terc^−/−^ mice (^*∗∗*^*P* ≤ 0.01), while nothing changed in WT mice. (d) There was also no significant difference among each subgroup for WT mice, and, compared with CON group, EA stimulation could significantly increase the D-value of frequency of locating platform between before and after treatment (^*∗*^*P* ≤ 0.05).

The results of the Morris water maze test are presented in Figure 2. In the hidden platform trial, the escape latency time in each group showed a downward trend in 4-day training time extension (Figures 2(a) and 2(b)). To analyze the effect of acupuncture treatment, the two probe trials were designed. The percentage of time spent in the target quadrant and the frequency of locating platform were used for statistical analysis. At the same time, the different values of the percentage of time and the frequency between pretreatment and posttreatment were further calculated to evaluate the significance of change after acupuncture. The results of probe trial showed that, compared with CON group, Terc^−/−^ mice in EA subgroup had significantly more variation to the time in quadrant where the platform used to be (*P* ≤ 0.01, Figure 2(c)), while there was almost no difference among three subgroups for WT mice. Meanwhile the D-value of frequency of locating platform between before and after treatment in EA group appeared significantly increased compared to CON group (*P* ≤ 0.05, Figure 2(d)), whereas the variation among WT mice shows no difference among three subgroups. These results demonstrated that EA acupuncture could ameliorate the cognitive deficits in the Terc^−/−^ mice, while it did not affect WT mice. Fortunately, these findings were consistent with our previous report [12].

### 3.2. Effects of EA Treatment Improved the Levels of TrkB Protein in the Hippocampus for Terc^−/−^ Group

**Figure 3:**
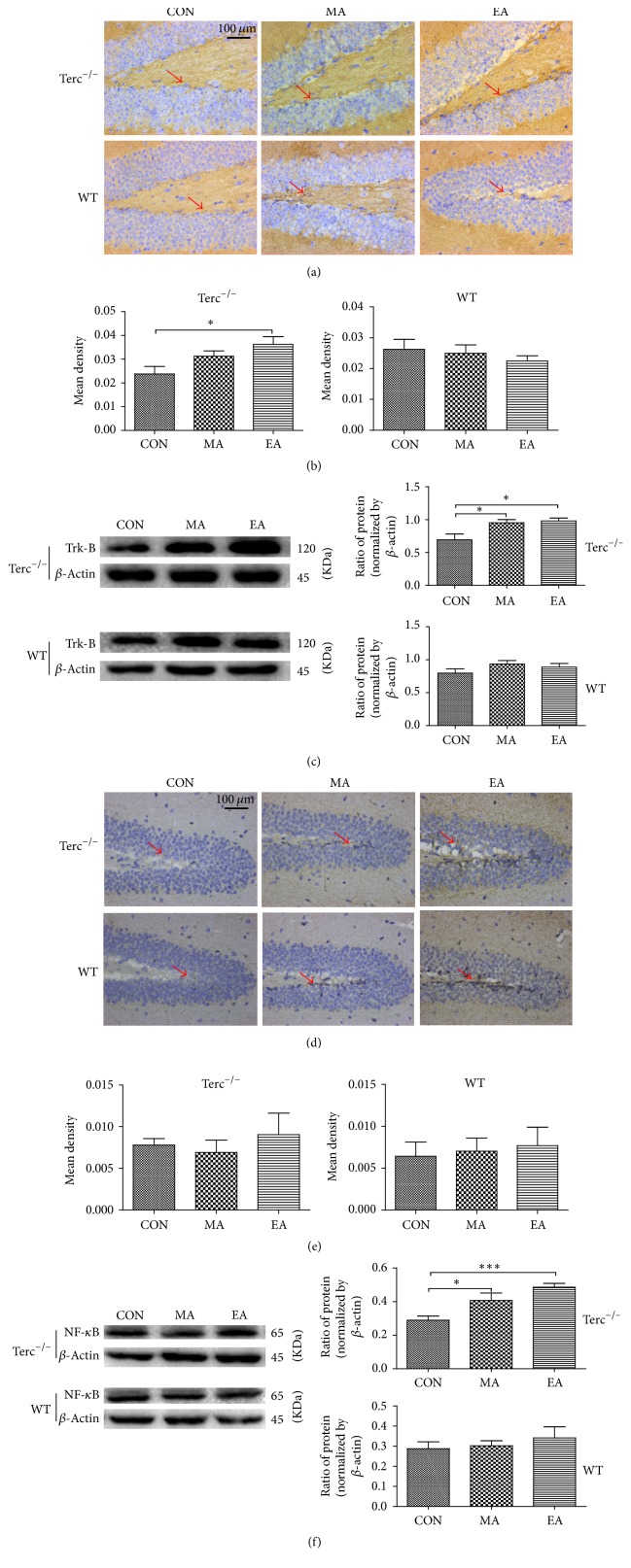
Immunohistochemistry and western blot analysis of TrkB/NF-*κ*B in hippocampus of the WT and Terc^−/−^ mice (*n* = 8 per subgroup). The brain samples were sliced sagittally into 5 *μ*m sections, and the representative photographs and the mean optical density of positive cell values are, respectively, shown in a and d; b and e. Data were expressed as mean ± SEM, and the data were analyzed using one-way ANOVA followed by Turkey's test of multiple comparisons. (a and d) The mice brain slices were hematoxylin-stained in the same region of the hippocampus among the three subgroups. The TrkB/NF-*κ*B positively stained cells appear brown (red arrow) in the subgranular zone (SGZ) around the dentate gyrus area (DG), and the scale bar = 100 *μ*m. (b) Compared with CON subgroup, the mean optical density of positive TrkB protein in EA subgroup was significantly increased in Terc^−/−^ mice (^*∗*^*P* ≤ 0.05), and there was no difference among the three subgroups for WT mice. Although there was obviously more NF-*κ*B positively strained cell observed in the picture, (e) no significantly differences were observed in NF-*κ*B immunoreactivity in any subgroups in both strains. (c and f) Expression of TrkB/NF-*κ*B in WT and Terc^−/−^ mice (*n* = 8 per subgroup) was detected by western blotting assay. Data are represented as the ratio of TrkB (NF-*κ*B)/*β*-actin. The bar graphs represent the levels of TrkB/NF-*κ*B in hippocampus in both strains. In the Terc^−/−^ mice, both electroacupuncture and manual acupuncture significantly increased the expression of TrkB/NF-*κ*B (^*∗*^*P* ≤ 0.05; ^*∗∗∗*^*P* ≤ 0.001), and nothing changed in WT mice.

Brain tissue samples from the subjects were analyzed using immunohistochemistry and western blot analysis to investigate the effect of acupuncture stimulation in the two strains of mice (Figures 3(a) and 3(c)). The TrkB protein is mainly distributed in the membrane in the subgranular zone (SGZ) around the dentate gyrus (DG) of the hippocampus. Even there were fewer weakly stains, they were still some positively stained cells. The result from the immunohistochemical evaluation indicated that only in Terc^−/−^ mice the stimulation of electroacupuncture (EA group) could significantly increase the expression of TrkB protein (^*∗*^*P* ≤ 0.05) compared with CON subgroup, and there was no obvious difference among any subgroup in WT mice (Figure 3(b)). The western blotting results of TrkB in the hippocampus were also shown that EA could promote the expression of TrkB compared with the other subgroup (Figure 3(c)), which was also consistent with our former reports [12].

### 3.3. Effects of EA Treatment Increased the NF-*κ*B Expression in the Hippocampus for Terc^−/−^ Group

To investigate whether EA or MA can alter the expression of TrkB downstream signal pathway, the expression of NF-*κ*B/ERK/p-ERK was measured in tissue sample. From the photograph, the positively stained NF-*κ*B appears brown mainly in the subgranular zone (SGZ) around the dentate gyrus area (DG) of hippocampus (Figures 3(d) and 3(e)), and there were no significant differences among any groups in the two strains of mice (Terc^−/−^ and WT mice). Meanwhile, the western blotting results of NF-*κ*B showed that, compared with CON group, the relative expressions of NF-*κ*B significantly increased in EA (^*∗∗∗*^*P* ≤ 0.001) and MA (^*∗*^*P* ≤ 0.05) subgroups for Terc^−/−^ mice (Figure 3(f)).

### 3.4. The Effects of Acupuncture Treatment on the Phosphorylation Levels of ERK

**Figure 4:**
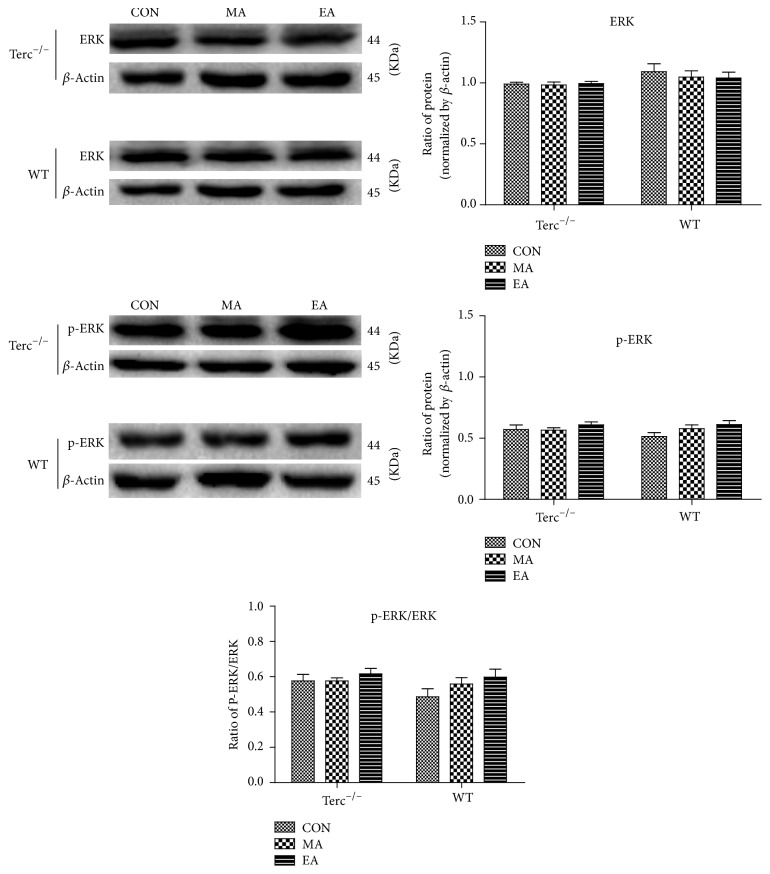
Expression level of ERK in the hippocampus of WT and Terc^−/−^ mice (*n* = 8 per subgroup) was detected by western blot assay. Blots were reprobed for expression of *β*-actin to control for loading and transfer. Data were expressed as mean ± SEM. The degree of ERK activation was represented as the ratio of p-ERK/ERK. The result demonstrated that the expression of ERK/p-ERK shows nothing significantly changed in the subgroups for the 2 types of mice, even in the ratio of p-ERK/ERK.

In order to further explore the mechanisms of acupuncture, the protein levels of p-ERK and ERK were measured by western blot to evaluate the activation of ERK. As p-ERK is a marker of ERK activation, the ratio of them was also calculated (Figure 4). The result demonstrated that neither electroacupuncture nor manual acupuncture showed significant differences in ERK/p-ERK expression among any subgroups in Terc^−/−^ mice (*P* ≥ 0.05) and likewise in the subgroups of WT mice. Furthermore, the ratio of p-ERK/ERK shows no significance in any subgroups for the 2 types of mice (*P* ≥ 0.05).

## 4. Discussion

As one of the most common tasks used to assess spatial learning and memory ability, the Morris water maze (MWM) was used in this study. The hidden platform trial and probe trial were used to assess the capabilities in spatial learning and memory, respectively. The abilities of spatial learning and memory were observed in the two strains of mice for 4 consecutive days [19]. The results of training period showed no significant difference among the various groups of mice, suggesting that all mice had the same learning and memory capacity before treatment (Figure 2).

Even the acupuncture has been widely applied for different kinds of nervous system, but there were still few studies that described whether the acupuncture intervention had different effects in different strains. In our present study, the Terc^−/−^ mice showed a better response to electroacupuncture. It implies the stimulation of acupuncture only produced therapeutic effects on animals at the pathological state. Some studies have reported that, in healthy animals [21], both electroacupuncture and manual acupuncture can lead to a significant increase in cell proliferation just in the SGZ of the DG. However, in our studies, only electroacupuncture can play a therapeutic role in the amelioration of learning and memory abilities for Terc^−/−^ mice [15, 22].

Recently, it has been reported that aging related neurodegenerative diseases are characterized by imbalance between neurogenesis and neurodegenerative diseases. And interestingly, some researches demonstrated that the stimulation of neurogenesis may be beneficial to patients with those diseases [23, 24]. In the current study, our research team found that, only in Terc^−/−^ mice, after acupuncture treatment, the TrkB/NF-*κ*B proteins were exhibited in the SGZ around the dentate gyrus area of hippocampus. We also found that EA administration showed more amelioration of reference memory impairment in Terc^−/−^ mice [2]. This suggests that EA administration alleviates aging risk by inhibiting reference memory decline. On the other hand, the hippocampus, which plays an important role in learning and memory, demonstrates a high degree of neurogenesis, and only the DG of hippocampus continues to develop through adulthood [25]. Presently more and more research demonstrated that there are only two neurogenic areas in the brain including subventricular zone (SVZ) of the lateral ventricles and the subgranular zone (SGZ) of the DG in hippocampus [26]. So it is obviously that the ability of undifferentiated and rapidly proliferating for the progenitor cells that could differentiate into granule in the SGZ of DG throughout life. In our study, the NF-*κ*B and TrkB positively strained cell could be found in SGZ area in hippocampus, and they showed significantly higher level compared with CON subgroup or MA subgroup. This indicates the electroacupuncture treatment can activate some protein signal pathways in the cell around DG for Terc^−/−^ mice. And the WT mice were not affected after the acupuncture stimulation [15, 27, 28].

For the difference between the effect of electroacupuncture and manual acupuncture, it is commonly accepted that EA stimulation shows a beneficial effect on neurodegeneration diseases. For manual acupuncture, the “De-Qi” feeling is essential to induce action. In clinical, acupuncture needles were repetitively penetrated up and down in different directions just for the purpose of “De-Qi” feeling [29, 30]. Consequently, some people believed that the effect of MA depends upon stimulating intensity (mild or strong) and duration of manipulation, even when the needle was tightly wound around by muscle fibers [31]. Some researches demonstrated that EA may cause electrical twitching of surrounding tissues and induce MA-like stimulation through mechanoreceptors [32]. The previous studies showed that manual acupuncture at ST36 significantly increased the number of BrdU-positive cells after ischemic injury [33, 34]. Subsequently, electroacupuncture stimulation at ST36 was reported to enhance cell proliferation in the DG a rat model of diabetes [9]. In our research, after electroacupuncture treatment, the hippocampal expression of TrkB was significantly increased in Terc^−/−^ group compared with the WT group. These results may indicate that the stimulation of acupuncture may have a close relationship with the neurogenesis in the hippocampus. For acupuncture, the acupoint was ST36, which is located on the anterior tibia muscle, and is one of the most important acupoints in clinical acupuncture for antiaging. Simulation of ST36 is carried out for a wide range of conditions affecting digestive system, cardiovascular system, immune system, and nervous system. Furthermore, ST36 is one of the seven acupoints used for stroke treatment. As the high-affinity BDNF receptor, the tyrosine protein kinase receptor B (TrkB) just the same as the BDNF was expressed in different kinds of neurons in the brain. [35–37]. Our previous studies demonstrated that manual acupuncture stimulation can activate the BDNF and its downstream signaling pathways [2].

Our study has shown that EA causes an increase in the positive cell of TrkB and NF-*κ*B signal pathway, but there was no evidence supporting that acupuncture can activate downstream protein of TrkB through ERK signal pathway. Several studies support that the activation of TrkB can prevent cell death by activating the ERK pathway in cortical neurons and cerebellar neurons [38]. And some researchers suggested that the ability of BDNF-TrkB to stimulate telomerase activity can be partially decreased through the total inhibition of the extracellular signal-regulated protein kinase (ERK) [6]. However, our study indicated that acupuncture can only specifically increase the expression of TrkB and NF-*κ*B in Terc^−/−^ mice instead of via the activation of ERK/p-ERK (Figures 3 and 4) protein. From the result, we found that even the ERK signal pathway plays an important role in the overall effects of electroacupuncture, but there was nothing changed in neurons of hippocampus [39]. As such, based on our result, it can be inferred that EA stimulation increased the ability of spatial learning and memory in Terc^−/−^ mice, and it might stem from the activation of NF-*κ*B.

Some studies demonstrated that NF-*κ*B is proinflammatory transcription factor which is increased in aging brain, and the activation of NF-*κ*B can protect neurons against death induced by neurodegeneration [40]. Therefore, the upregulated of NF-*κ*B could show an neuroprotective effect in our brain [41]. At the same time, reactive oxygen species (ROS) have been implicated in many aspects of aging and in neurodegenerative diseases [42]. And NF-*κ*B is oxygen sensitive and also is a precursor to VEGF (vascular endothelial growth factor) gene expression that leads to angiogenesis, it can regulate the proinflammatory response in endothelial cells [43]. Several studies supported that the role of NF-*κ*B depends on the types of axoneuron, and the activation of NF-*κ*B in ischemic dementia caused the neuron degeneration to microglia in cortex. However the neuroprotection effect was shown in the hippocampal neuron cell [43–45]. Our result indicated that NF-*κ*B could be specifically increased by electroacupuncture in Terc^−/−^ mice rather than WT mice, and the positive cells were exhibited in the SGZ around the dentate gyrus area of hippocampus. It suggests that the electroacupuncture may be involved in the nerve regeneration in SGZ; furthermore the positive increasing expression of TrkB and NF-*κ*B in the subgranular zone (SGZ) around the dentate gyrus (DG) area may be a possible mechanism of EA in the treatment of aging in telomerase-deficient mice.

## 5. Conclusions

In summary, our key findings suggest that, compared with MA, the application of EA could ameliorate the spatial learning and memory ability for telomerase-deficient mice; furthermore, it could also increase the expression of TrkB and NF-*κ*B in the subgranular zone (SGZ) around the dentate gyrus (DG) area. Based on this result, it is also suggested that the neuroprotection and neuron regeneration may play a critical role in electroacupuncture-induced antiaging effect. At the same time, the mechanisms of EA and MA effects on telomerase-deficient mice further provide the theoretical basis for antiaging clinical applications.
